# Supplementary figures and images for: Tracking progress towards malaria elimination in China: Individual-level estimates of transmission and its spatiotemporal variation using a diffusion network approach
Source: PLoS Comput Biol. 2020 Mar 23;16(3):e1007707. doi: 10.1371/journal.pcbi.1007707 (PMC7117777; doi:10.1371/journal.pcbi.1007707)

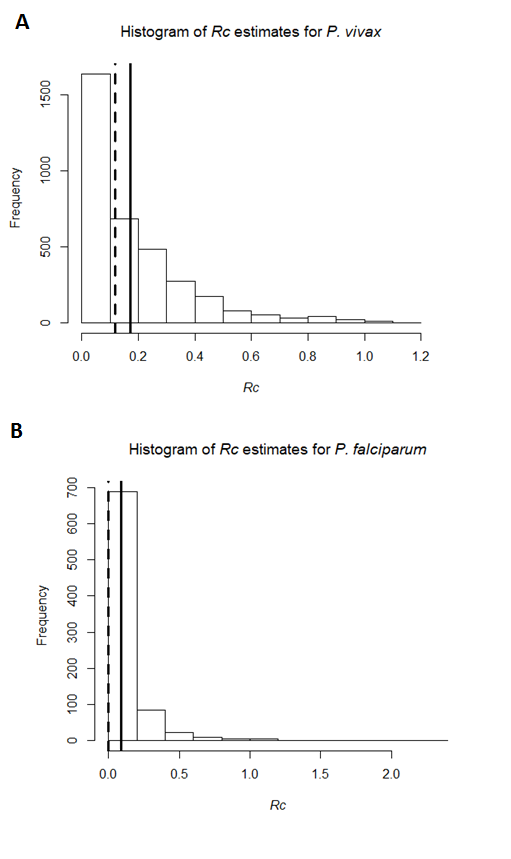

Supplement: S1 Fig — Histogram of Rc estimates for A) P. vivax in Yunnan and B) P. falciparum in Yunnan. Dotted lines show median, solid lines show mean. (PNG) [file pcbi.1007707.s001.png]

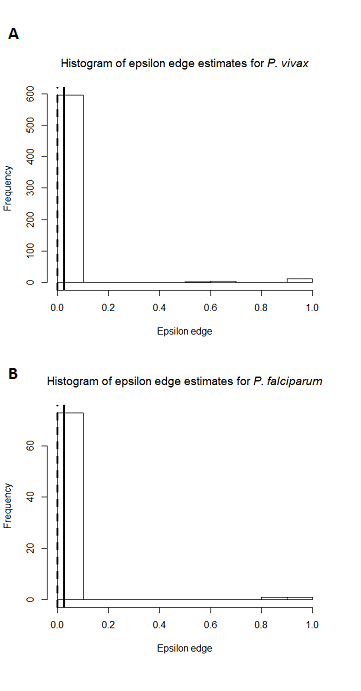

Supplement: S4 Fig — Histogram of epsilon edge distribution for A) P. vivax, B) P. falciparum. (PNG) [file pcbi.1007707.s004.png]

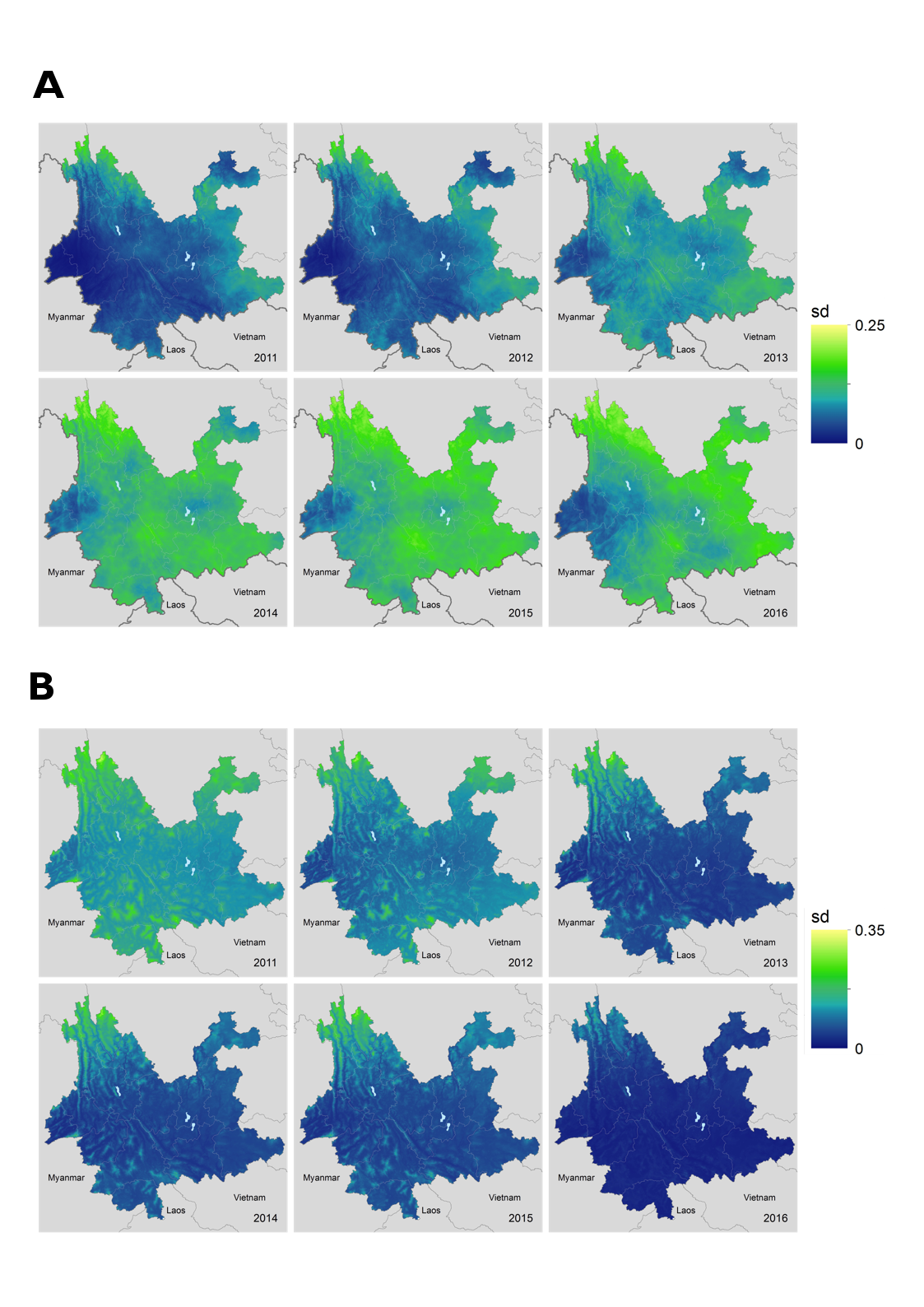

Supplement: S5 Fig — Map of standard deviation in estimates of P(Rc >0) for A) Plasmodium falciparum and B) Plasmodium vivax. (PNG) [file pcbi.1007707.s005.png]

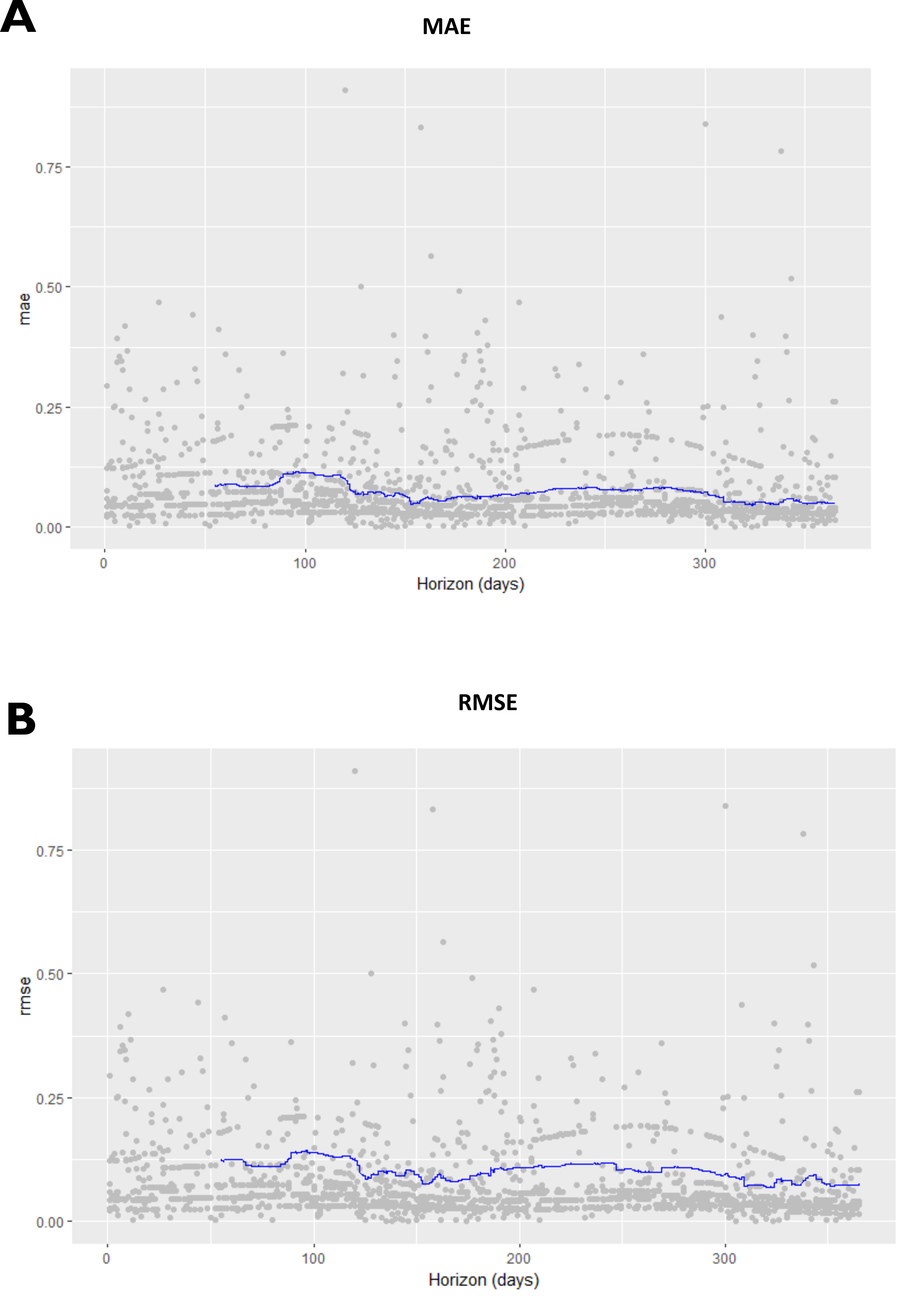

Supplement: S6 Fig — Plots show A) Root Mean Squared Error (RMSE) B) Mean Absolute Error (MAE). The training set used was the first 730 days of data and the horizon used was 365 days. (PNG) [file pcbi.1007707.s006.png]

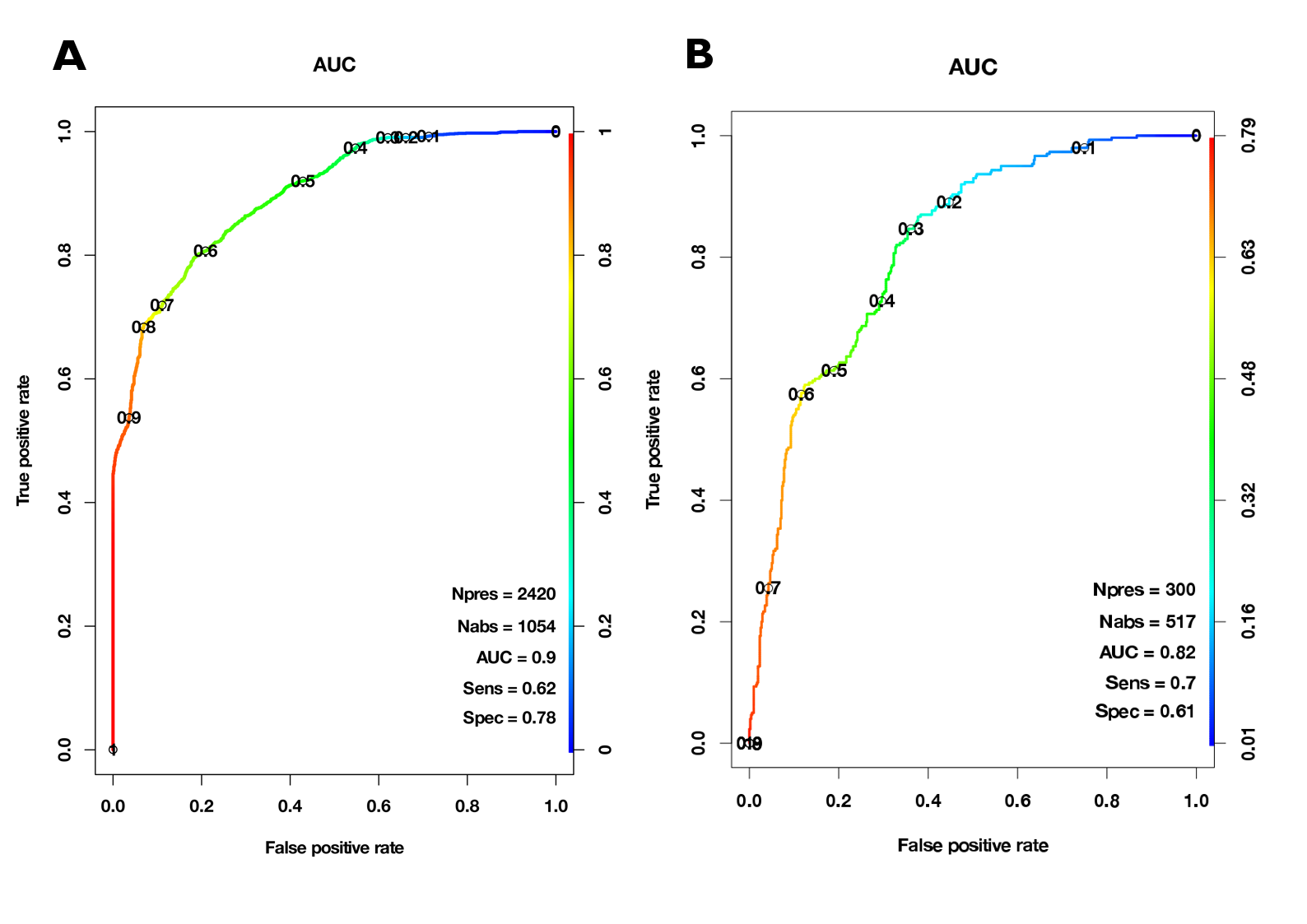

Supplement: S7 Fig — ROC curve plot for map of P(Rc risk >0) for A) P. vivax B) P. falciparum. (PNG) [file pcbi.1007707.s007.png]
