# Supplementary material for: Tracking progress towards malaria elimination in China: Individual-level estimates of transmission and its spatiotemporal variation using a diffusion network approach
Source: PLoS Comput Biol. 2020 Mar 23;16(3):e1007707. doi: 10.1371/journal.pcbi.1007707 (PMC7117777; doi:10.1371/journal.pcbi.1007707)
Supplement: S1 Text — (DOCX) [file pcbi.1007707.s008.docx]

### S1 Text: Surveillance system in China

###

China has a sophisticated malaria surveillance system, described in detail elsewhere (1–5). Briefly, surveillance is carried out in both a passive and reactive manner, organised and administered at the national, provincial and county level. The centralised China Information System for Disease Control and Prevention (CISDCP) receives daily updates on case reports from health facilities

Passive detection occurs according to a protocol at the local level, such that cases are tested by microscopy or Rapid Diagnostic Test (RDT) and reported to the central information system within 24 hours. Case investigation is then pursued, where cases are confirmed via double readings of microscopy slides and in some cases polymerase chain reaction (PCR) confirmation at provincial laboratories. At this point it is also determined whether the case is locally acquired or imported by taking patient travel history – if a patient has travelled to a malaria endemic country within a month of symptom onset the case is then classified as imported (3). Case investigation should be completed within three days.

Foci investigation occurs once a case is detected to determine whether the foci is inactive, active or a pseudo-focus based upon the absence or presence of suitable vectors (inactive), and presence or absence of malaria in the resident area of the case if imported (pseudo-focus). Reactive Case Detection (RACD) of case contacts and populations with demographic similarities (for example individuals working in the same industry and vicinity as the case) is carried out. In active foci more intensive RACD screening of a larger pool of neighbours and contacts is carried out using Rapid Diagnostic Tests (RDTs) for immediate detection, followed by PCR testing of blood spots to detect low-density infections. IRS (Indoor Residual Spraying) is also carried out(1,3,5).

The Ministry of Health (MoH) in China has also been measuring the timeliness of the recommended protocol and follow-on ability to meet these targets. It was found that the one-day target for case reporting was almost always met because this is required by law**.** In the years following the introduction of the 1-3-7 policy, the proportion of cases investigated within three days increased from roughly 55% in 2011 to almost 100% by 2013. However the programme took longer to achieve the seven day focal point investigation goals, with just over 50% of foci investigated and treated within seven days by the end of 2013 (3). Nevertheless, by 2015, adherence to the 1-3-7 strategy improved and this figure increased to an estimated 96% (5)**.** Whilst some cases could still be missed, the thoroughness of the approach means numbers of missing cases are likely to be small.

References

1. Feng X-Y, Xia Z-G, Vong S, Yang W-Z, Zhou S-S. Surveillance and Response to Drive the National Malaria Elimination Program. Adv Parasitol [Internet]. 2014 Jan 1 [cited 2018 Nov 15];86:81–108. Available from: https://www.sciencedirect.com/science/article/pii/B9780128008690000044?via%3Dihub

2. Hu T, Liu YB, Zhang S Sen, Xia ZG, Zhou S Sen, Yan J, et al. Shrinking the malaria map in China: Measuring the progress of the National Malaria Elimination Programme. Infect Dis Poverty. 2016;5(1).

3. Cao J, Sturrock HJW, Cotter C, Zhou S, Zhou H, Liu Y, et al. Communicating and Monitoring Surveillance and Response Activities for Malaria Elimination: China’s “1-3-7” Strategy. PLoS Med [Internet]. 2014 May [cited 2018 Nov 21];11(5):e1001642. Available from: http://www.ncbi.nlm.nih.gov/pubmed/24824170

4. Yang GJ, Tanner M, Utzinger J, Malone JB, Bergquist R, Yy Chan E, et al. Malaria surveillance-response strategies in different transmission zones of the People’s Republic of China: Preparing for climate change. Malar J. 2012;

5. Zhou S-S, Zhang S-S, Zhang L, Rietveld AEC, Ramsay AR, Zachariah R, et al. China’s 1-3-7 surveillance and response strategy for malaria elimination: Is case reporting, investigation and foci response happening according to plan? Infect Dis Poverty [Internet]. 2015 Dec 10 [cited 2019 Jan 10];4(1):55. Available from: http://idpjournal.biomedcentral.com/articles/10.1186/s40249-015-0089-2
