# Supplementary material for: Tracking progress towards malaria elimination in China: Individual-level estimates of transmission and its spatiotemporal variation using a diffusion network approach
Source: PLoS Comput Biol. 2020 Mar 23;16(3):e1007707. doi: 10.1371/journal.pcbi.1007707 (PMC7117777; doi:10.1371/journal.pcbi.1007707)
Supplement: S2 Text — (DOCX) [file pcbi.1007707.s009.docx]

# Supplementary Note 2: Tests of algorithm on simulated data

Simulations were carried out to explore the impact of various assumptions on the ability of the model to recover correct reproduction number estimates and serial intervals. Two approaches were used: firstly, simulating epidemics along explicit networks using a network based susceptible-infected model, and secondly using a stochastic SIR model with a given $R_{c}$ distribution to simulate line lists.

## Simulation across networks

For the first simulation, data were simulated by generating small-world networks using the *igraph* (1) package in R version 3.3 (2). Small world networks are hypothesised to reflect many real-life networks, which show both properties of regularity and randomness(3,4). The network generated for this analysis is illustrated in figure SN1. Then a susceptible-infected (SI) model was run along the network, where during each time step infected nodes infect their neighbours with probability β.

Under the SI model, at time zero $(t=0)$, all nodes begin susceptible, bar a given number of seed nodes. For this simulation, initially one node was seeded with infection. At $t=1$ the infected node can infect each neighbouring node which shares an edge with it (determined by the simulated network), with probability β. For this simulation β is constant, provided the infected node is connected by an edge to a susceptible node. At $t=2$if any new infections occur, the newly infected nodes then become able to infect their neighbours with probability β. The chain continues for a set horizon of time or until all nodes are infected. The incidence time series generated by this simulation was then input into a frequentist version of the algorithm.

Two factors were measured to explore the accuracy and effectiveness of the algorithm. Firstly, the mean $\alpha_{ij}$value returned by the model, which is defined as the instantaneous hazard of infection, which for an exponential parameterisation is not time dependent. The true alpha value was assumed to be β, the hazard of infecting neighbours. Secondly, the corresponding functions determining likelihood of transmission were also compared.


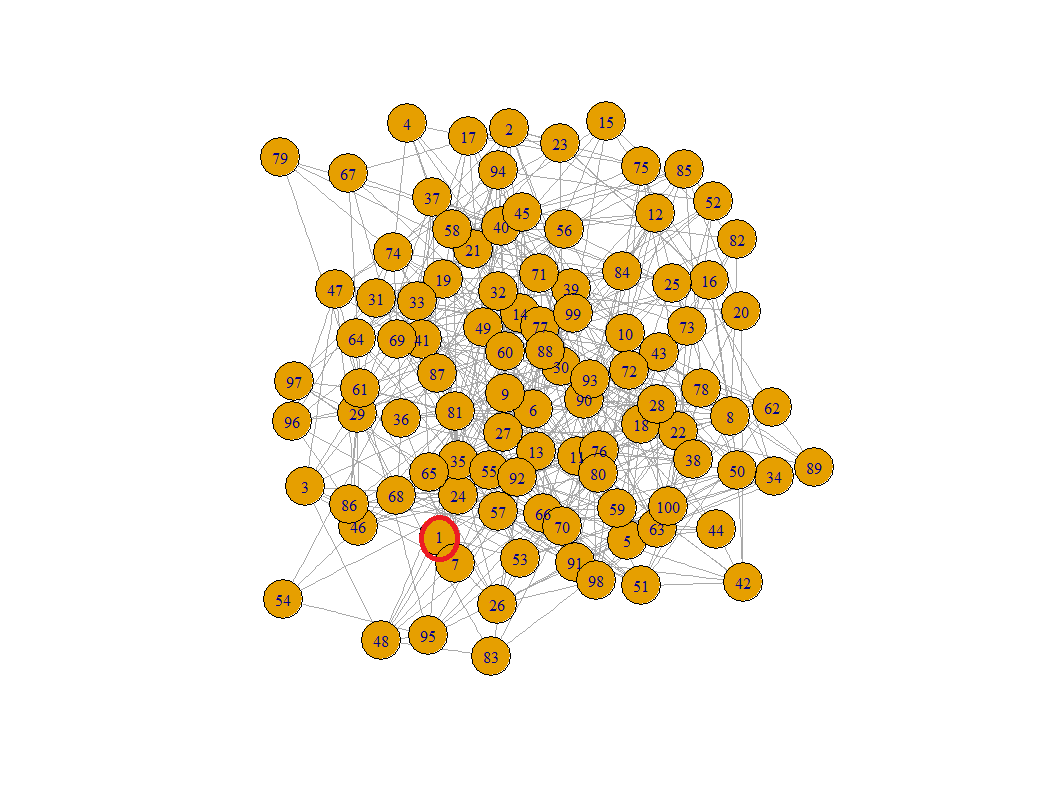


Figure SN1: Network used for simulation. Note the edges here represent potential connections and routes along which transmission could occur. Node 1, circled in red always seeded transmission.

## Stochastic SIR simulation of line lists

To further test assumptions in model we simulated line lists with missing data using *EpiGenR*, an algorithm and R package which simulates transmission events and then samples from this to represent a final detected line list. This model implements a stochastic Susceptible-Infected-Recovered model over discrete time steps in the C++ language via the *Rcpp* package. Recovery is exponentially distributed, with rate parameter,$\gamma$. This parameter determines the time to infection of the next generation and in turn the serial interval distribution. Infectors infect a number of individuals, drawn from the offspring or reproductive number distribution, which is negative binomial with dispersion parameter $K.$

To reflect an elimination scenario, the distribution of individual reproduction numbers was defined as a negative binomial distribution with mean 0.5 considered two values for the overdispersion parameter, $K$, as 0.1 (more overdispersed, more variance in $R_{c}$) and 1 (less overdispersed, less variance in $R_{c}$). We then, for both values of $K$, simulated 100 outbreaks of minimum infected size 100 over 1000 days, with an exponentially distributed serial interval with a mean of 30 days and measured the ability of the algorithm to detect the underlying offspring distribution. Each outbreak had 100 seed infectors in a fully susceptible population of 50 000, with no further importation occurring, to ensure the final sample size was large enough to measure $R_{c}$. As the simulator draws integers, for better comparison of model estimated results, the distribution of $R_{c}$ estimates where each maximum-a-posteriori estimate is rounded to the nearest integer as well as the raw estimates. Both the histograms and means of simulated versus estimated results were compared.

To simulate missing cases, the fully observed dataset was sampled following a proportional approach where for each case the probability of observation was set at varying values between 1 and 0.3, and then each individual observation was determined by drawing from a binomial distribution with the given probability.

## Simulation Results

Simulated data on a small world network found method inferred mean $\alpha_{i,j}$, or instantaneous hazards of transmission to be relatively similar to expected values, as shown in Figure SN 2. The corresponding likelihood of infection also closely resembled the true likelihood (Figure SN 3), assuming the same parametric form (an exponentially distributed likelihood, determined by $\alpha_{ij}$and time).


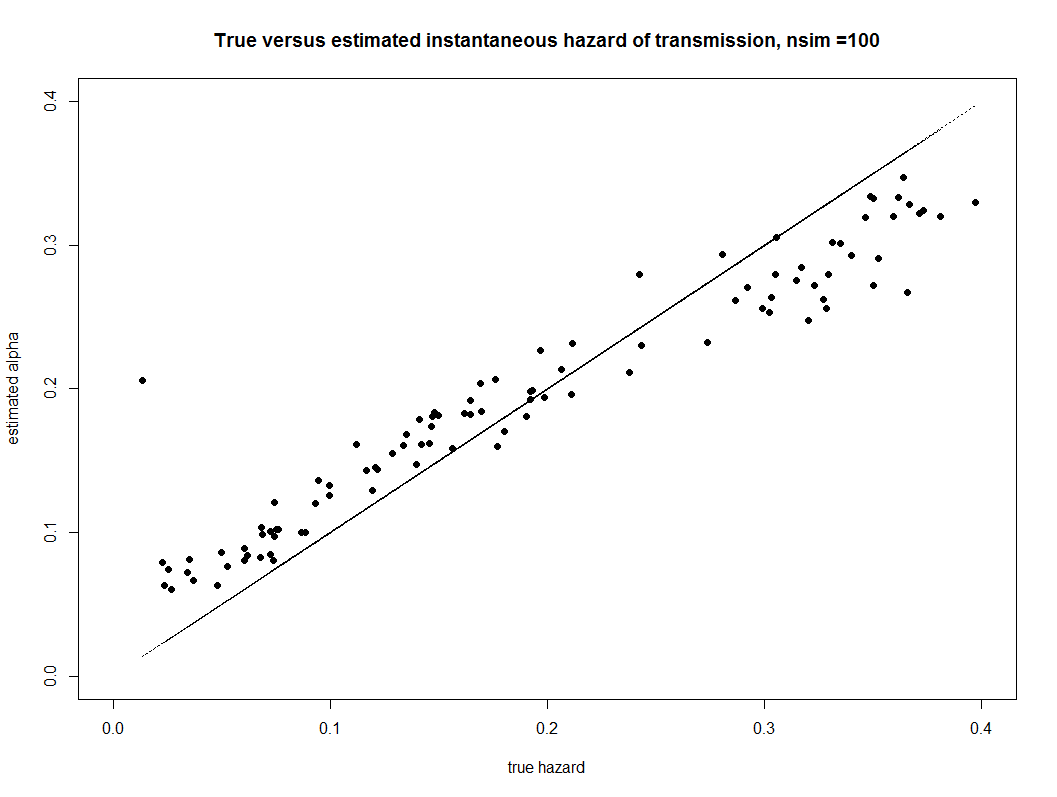


Figure SN 2 Plot of true transmission rate plotted against model estimated transmission rate (hazard)


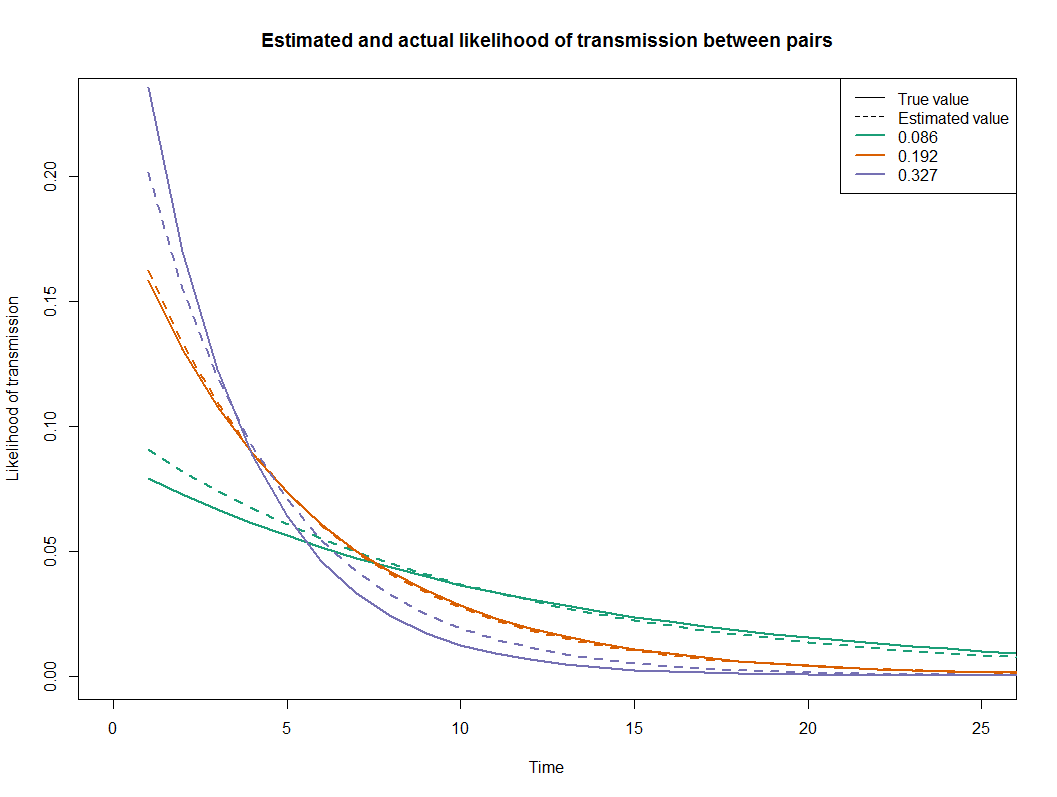


Figure SN 3: Three randomly drawn estimated and actual alpha values from figure SN2, showing the corresponding estimated and actual transmission likelihoods they represent. Colours show likelihoods of transmission over time for different values of actual alpha(solid line) and their corresponding estimated values (dotted line).

## Simulation using stochastic SIR model

#### Simulations from a more overdispersed R distribution (K=0.1)

When the probability of observing a case was 1, $P\left( case observed \right)=1$, simulated linelists, simulated from a negative binomial $R_{c}$ distribution of mean ($\mu$) 0.5, with overdispersion parameter $(K)$, of 0.1 ( $R_{c}\sim Negative Binomial( \mu=0.5, K=0.1$)) had a true mean $R_{c}$ of 0.56. When the prior for the $\varepsilon$edge was defined as having a Truncated Normal prior with mean = 0.001 and standard deviation = 1, ($prior\left( \varepsilon\right)\sim Truncated Normal( \mu=0.001, =1)$) the algorithm returned a mean of 0.54 when results were rounded to the nearest integer and 0.6 when decimal values were not rounded (figures SN 4C). When the probability of observation was 90%, this value decreases to 0.52 (rounded) and 0.56 (decimal). The mean estimate continues to decrease with decreasing observations (figures SN 5-7), but even with an average of 30% of cases observed, the mean $R_{c}$ was estimated as 0.41 and 0.46 when $R_{c}$ is a rounded integer or decimal estimate respectively (figure SN7).

#### Simulations from a less overdispersed R distribution (K=1)

When the probability of observing a case was 1, $P\left( case observed \right)=1$, line-lists, simulated from a negative binomial $R_{c}$ distribution of mean ($\mu$) 0.5, with overdispersion parameter $(K)$, of 1 ( $R_{c}\sim Negative Binomial( \mu=0.5, K=1$)) had a true mean $R_{c}$ of 0.59. When the prior for the $\varepsilon$edge was defined as having a Truncated Normal prior with mean = 0.00001 and standard deviation = 1, ($prior\left( \varepsilon\right)\sim Truncated Normal( \mu=0.00001, K=1)$) , the algorithm returned a mean of 0.54 when results were rounded to the nearest integer and 0.53 when decimal values were not rounded (figures SN8). When the probability of observing a case was 0.9 ($P\left( case observed \right)=0.9)$, this value decreases to 0.49 (rounded) and 0.52 (decimal). When an accurate and informative prior for $\varepsilon$ when ($P\left( case observed \right)=0.9)$ is chosen, the model accurately returns the mean $R_{c}$ of 0.59 (Fig. SN9). With an average of 30% of cases observed, the mean $R_{c}$ was estimated as 0.41 and 0.44 when $R_{c}$ is a rounded integer or decimal estimate respectively. Observationally, the distribution of $R_{c}$s remain similar to the true value (Fig SN10), however more quantitative analysis would be required to rigorously assess similarities in the distributions.

**A**

**B**

**C**


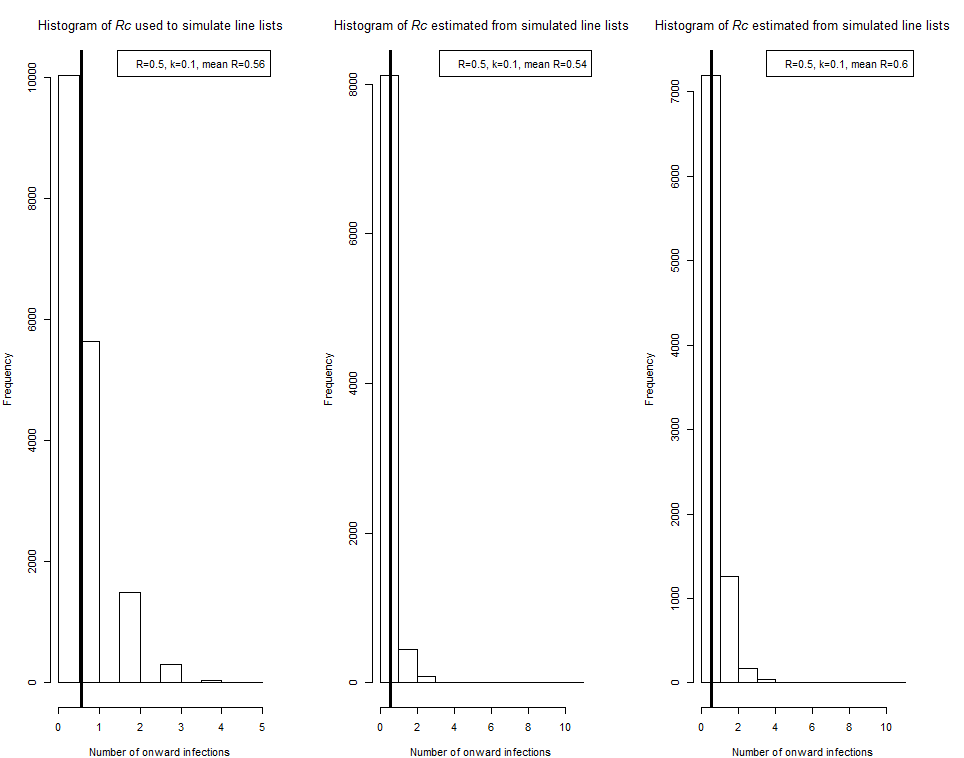


Figure SN4: When P(case observed) = 1.0. : A) Histogram of individual reproduction numbers from simulated data, B) model estimated results when estimates rounded to the nearest integer and C) when decimal estimates are used


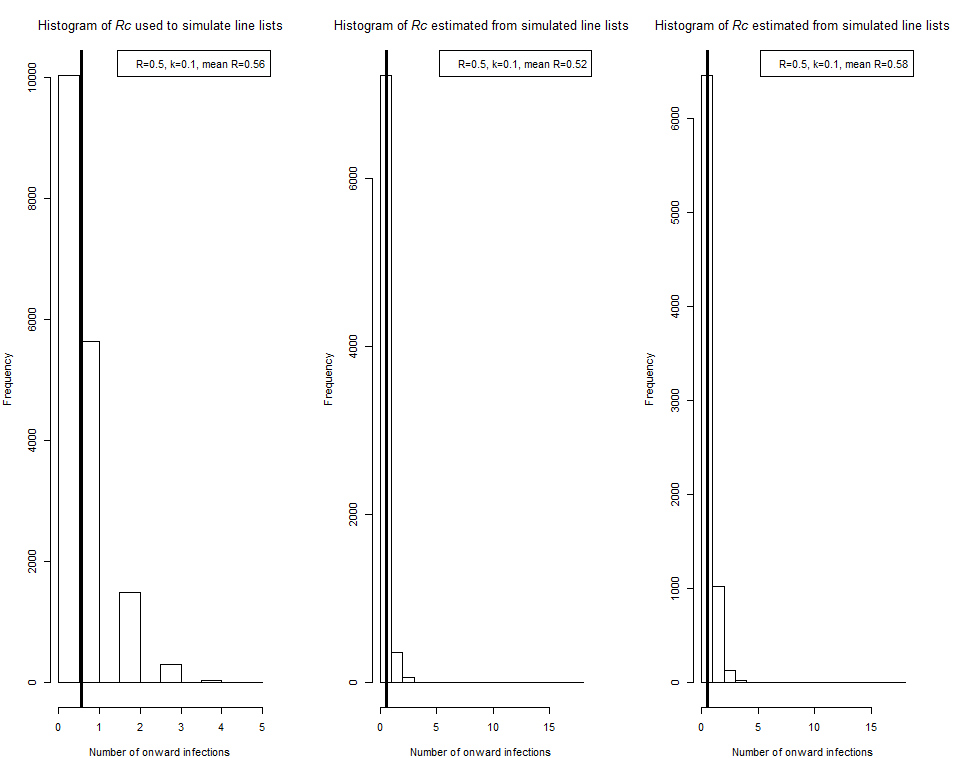


**A**

**B**

**C**

Figure SN5: When P(case observed) = 0.9. : A) Histogram of individual reproduction numbers from simulated data, B) model estimated results when estimates rounded to the nearest integer and C) when decimal estimates are used


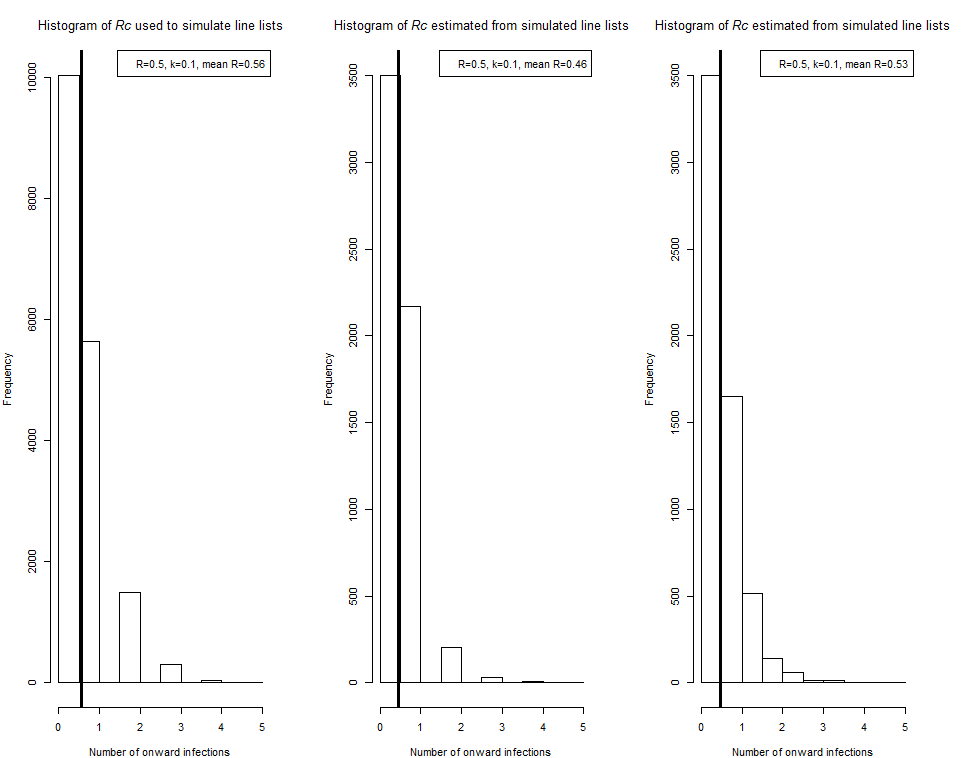


**A**

**B**

**C**

Figure SN6: When P( case observed) = 0.7. A) Histogram of individual reproduction numbers from simulated data, B) model estimated results when estimates rounded to the nearest integer and C) when decimal estimates are used

Figure SN6: When P(case observed) = 0.7. A) Histogram of individual reproduction numbers from simulated data, B) model estimated results when estimates rounded to the nearest integer and C) when decimal estimates are used


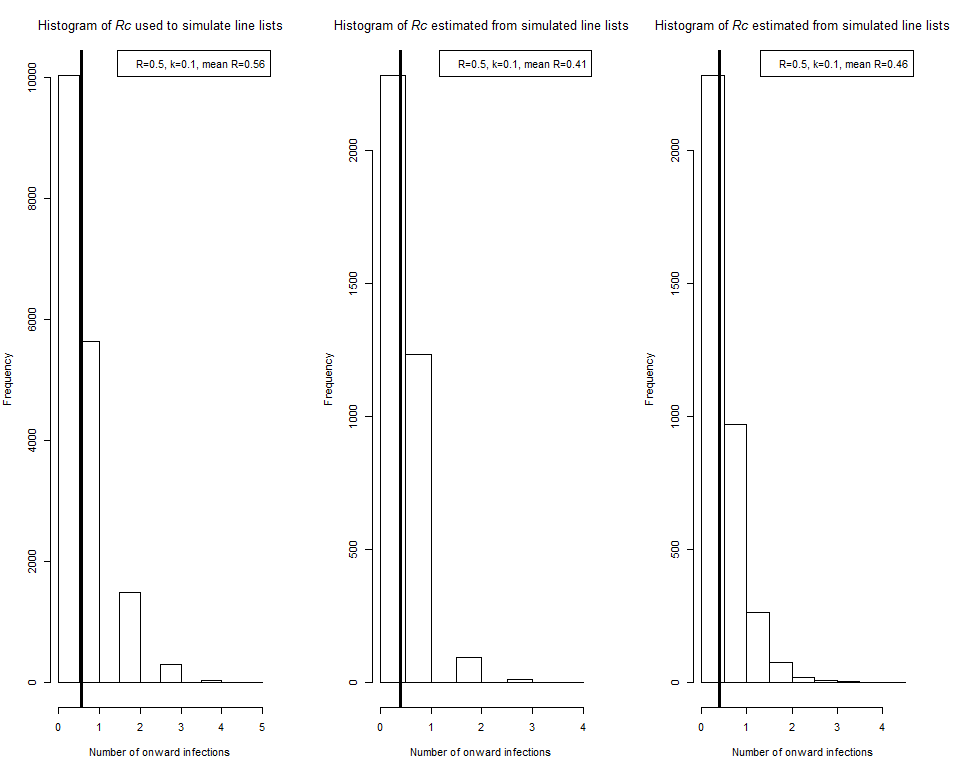


**A**

**B**

**C**

Figure SN7: When P(case observed) = 0.3: A) Histogram of individual reproduction numbers from simulated data, B) model estimated results when estimates rounded to the nearest integer and C) when decimal estimates are used.


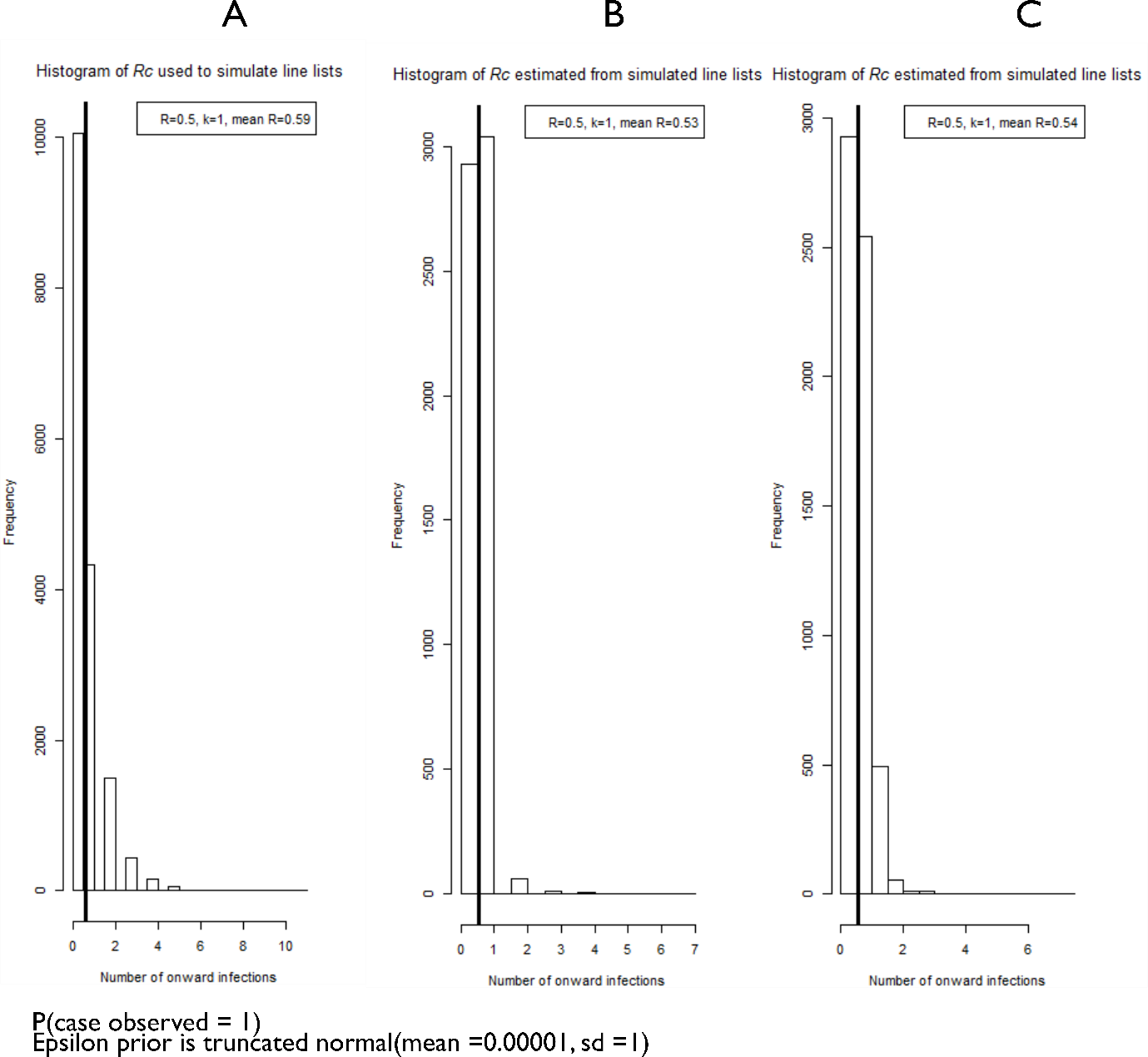


Figure SN8: When P(case observed) = 1.0 and an uninformative prior used for $\varepsilon(TruncatedNormal\left( mean=0.0001, standard deviation=1 \right).$ A) Histogram of individual reproduction numbers from simulated data, B) model estimated results when estimates rounded to the nearest integer and C) when decimal estimates are used.


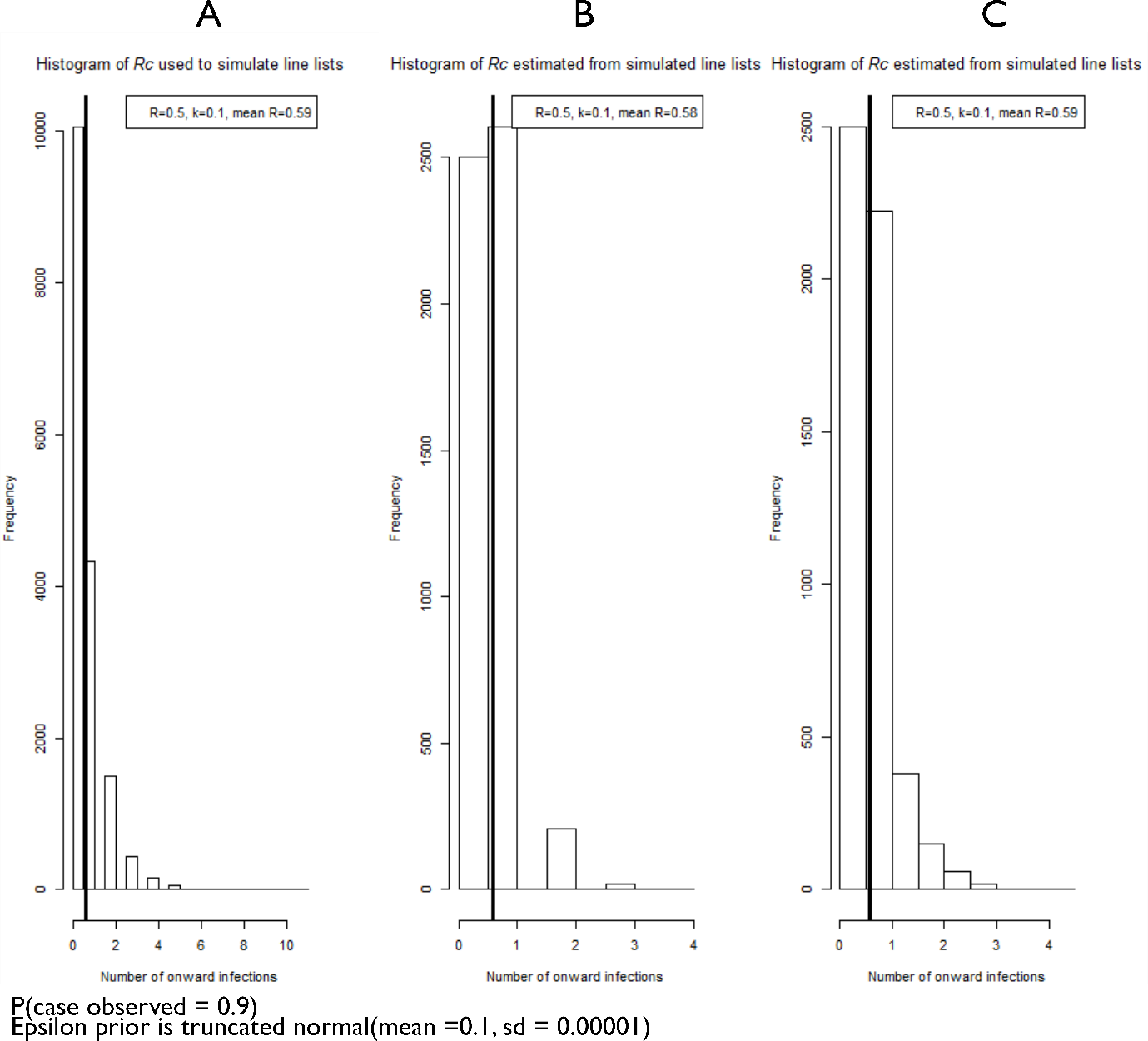


Figure SN9: When P(case observed) = 0.9 and informative and accurate prior used for $\varepsilon, (TruncatedNormal(mean=0.1, standard deviation=0.00001)$ A) Histogram of individual reproduction numbers from simulated data, B) model estimated results when estimates rounded to the nearest integer and C) when decimal estimates are used.


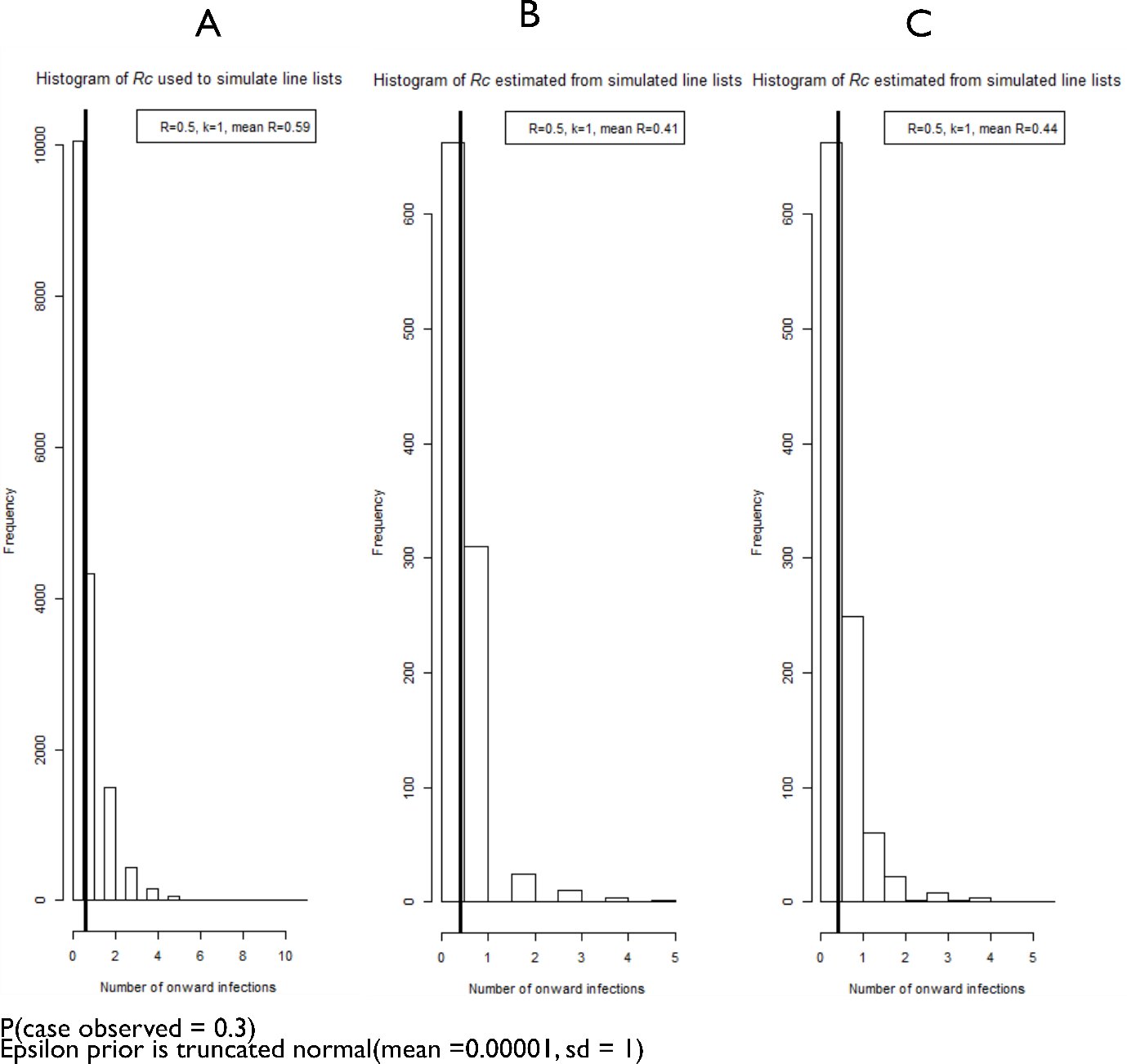


Figure SN10: When P(case observed) = 0.9 and an uninformative prior used for $\varepsilon(TruncatedNormal\left( mean=0.0001, standard deviation=1 \right).$ A) Histogram of individual reproduction numbers from simulated data, B) model estimated results when estimates rounded to the nearest integer and C) when decimal estimates are used.

# References

1. Csardi G, Nepusz T. The igraph software package for complex network research. InterJournal [Internet]. 2006;Complex Sy:1695. Available from: http://igraph.sf.net

2. R Core Team. R: A Language and Environment for Statistical Computing [Internet]. Vienna, Austria; 2016. Available from: https://www.r-project.org/

3. Watts D, Strogatz S. Collective dynamics of’small-world’networks. Nature [Internet]. 1998 [cited 2017 Jun 2]; Available from: http://search.proquest.com/openview/afbb88ac45f3437067fbc694e38687a3/1?pq-origsite=gscholar&cbl=40569

4. Eubank S, Guclu H, Anil Kumar VS, Marathe M V., Srinivasan A, Toroczkai Z, et al. Modelling disease outbreaks in realistic urban social networks. Nature [Internet]. 2004 May 13 [cited 2016 Jan 8];429(6988):180–4. Available from: http://dx.doi.org/10.1038/nature02541
