# Supplementary material for: Tracking progress towards malaria elimination in China: Individual-level estimates of transmission and its spatiotemporal variation using a diffusion network approach
Source: PLoS Comput Biol. 2020 Mar 23;16(3):e1007707. doi: 10.1371/journal.pcbi.1007707 (PMC7117777; doi:10.1371/journal.pcbi.1007707)
Supplement: S1 Table — A) Cases by diagnosis type (probable and confirmed) and species across China. B) Cases by imported/local status and species across China. C) Cases by diagnosis type (probable and confirmed) and species across Yunnan Province. D) Cases by imported/local status and species across Yunnan province2 (DOCX) [file pcbi.1007707.s010.docx]

### S1A Table: Cases by diagnosis type (probable and confirmed) and species across China

|  |  |  |  |  |  |  |
| --- | --- | --- | --- | --- | --- | --- |
|  | **Mixed infection** | ***P. falciparum*** | ***P. malariae*** | ***P. ovale*** | ***P. vivax*** | **Untyped** |
| **Confirmed** | 260 | 11830 | 252 | 822 | 6631 | 87 |
| **Probable** | 0 | 176 | 0 | 0 | 693 | 311 |
|  |  |  |  |  |  |  |

### S1B Table: Cases by imported/local status and species across China

|  | **Mixed infection** | ***P. falciparum*** | ***P. malariae*** | ***P. ovale*** | ***P. vivax*** | **Untyped** |
| --- | --- | --- | --- | --- | --- | --- |
| **Local** | 5 | 92 | 4 | 1 | 1711 | 95 |
| **Imported** | 255 | 11914 | 248 | 821 | 5613 | 303 |

### S1C Table: Cases by diagnosis type (probable and confirmed) and species across Yunnan Province

|  |  |  |  |  |  |  |
| --- | --- | --- | --- | --- | --- | --- |
|  | **Mixed infection** | ***P. falciparum*** | ***P. malariae*** | ***P. ovale*** | ***P. vivax*** | **Untyped** |
| **Confirmed** | 27 | 770 | 8 | 1 | 3269 | 3 |
| **Probable** | 0 | 21 | 0 | 0 | 200 | 64 |
|  |  |  |  |  |  |  |

### S1D Table: Cases by imported/local status and species across Yunnan province

|  |  |  |  |  |  |  |
| --- | --- | --- | --- | --- | --- | --- |
|  | **Mixed infection** | ***P. falciparum*** | ***P. malariae*** | ***P. ovale*** | ***P. vivax*** | **Untyped** |
| **Local** | 4 | 71 | 0 | 0 | 611 | 51 |
| **Imported** | 23 | 720 | 8 | 1 | 2658 | 16 |
