# Supplementary material for: Tracking progress towards malaria elimination in China: Individual-level estimates of transmission and its spatiotemporal variation using a diffusion network approach
Source: PLoS Comput Biol. 2020 Mar 23;16(3):e1007707. doi: 10.1371/journal.pcbi.1007707 (PMC7117777; doi:10.1371/journal.pcbi.1007707)
Supplement: Attachment — Submitted filename: Response_reviewers_Routledgeetal.docx [file pcbi.1007707.s012.docx]

Dear Reviewers,

Thank you for your constructive comments and suggestions. They have been very helpful and have improved our manuscript. I have included our responses to each specific reviewer comment below, with our responses italicised below each comment. I also attach a version of the edited manuscript with the tracked changes which we have made in response to your suggestions.

**Reviewer #1**

**Major compulsory comments to be addressed:
• The evidence for lack of a P. vivax relapse is stated to be from the surveillance dataset as each patient is given a unique identifier. I would argue this does not preclude there to be a fair number of relapsed P. vivax cases. How would this have influenced the estimation of Rc? I’m assuming it would have had limited impact on these estimates, but the authors should discuss this in the discussion.**

*The issue of relapse is an important point. It is worth noting that in addition to searching the individual based electronic data for relapse and finding no evidence of this, there are several other features of malaria control in China that also provide evidence that it is unlikely that a large proportion of the observed cases are due to relapse. According to China's National Malaria Elimination Technology Program (2011), the epidemiological history of each case has been investigated to check the source of the infection and the history of previous infection and relapse malaria. In addition, all malaria cases received free antimalarial treatment, and each case of P. vivax malaria was treated with radical cure.*

*Nonetheless, there is a chance of some relapse malaria which was missed during case investigation, treatment and surveillance. In our approach we jointly estimate unobserved sources of infection but are agnostic as to the specific cause of the unobserved source. As a result, relapses are considered as one of the potential unobserved sources of infection. Although large amounts of relapse are unlikely for the reasons outlined above, it is true that if there were very large amounts of relapse, the estimated reproduction numbers could be overestimated. However, given that we find such low reproduction numbers, even if this unlikely situation were the case, this does not impact our key findings and in fact would be stronger evidence of China achieving strong reductions in malaria transmission. I have added a discussion point about relapse in lines 259-272 in the discussion section.*

**• The authors argue there are a limited number of unobserved cases in the population because of strong reporting, investigation and response. What about treatment seeking in the community, where even with mild/non-severe symptoms, an individual** **either does not seek treatment, or finds alterative treatment though self-medicating or through informal providers? Are there any data on treatment seeking for fevers in Yunnan? I would be particularly concerned if lack of treatment seeking were also associated with certain populations that would be more likely to travel to malaria endemic settings, such as migrants or undocumented immigrants, who also might be at increased risk of infection and importation (and being an unobserved case). How might such unobserved cases have impact spatio-temporal trends in Rc? This would also be good to bring up in the discussion.**

*Yes, another important point to consider and discuss.* *Yunnan has achieved full coverage of basic healthcare at village/community level, and local residents and migrants can easily and timely access to health services. According to the National Malaria Elimination Action Plan, hospitals/CDCs provide free antimalarials to cure both local and imported malaria cases found in Chinese residents, mobile populations or foreigners, and pharmacies and non-medical institutions cannot sell antimalarials. Additionally, since China launched the Malaria Elimination Action Plan in 2010, Yunnan Province has special fund to support enhanced malaria surveillance with a high sensitivity, as it was identified as a key province with endemic malaria.  The healthcare units at township level are required to screen malaria in febrile patients, so that the possibility of missed diagnosis or underreports of malaria in the patients with fever is likely to be low. Due to the low levels of malaria in China, and higher but still low transmission contexts of most of the countries of origin of P.vivax importation in Yunnan (Myanmar), immunity is likely to be low and therefore asyptomatic infection is less likely. For those who do have no fever or mild symptoms, we assume they have limited capacity to transmit malaria as their gametocyte density is likely to be low. Although there has been work demonstrating the ability for asymptomatic infection to contribute to onward transmission, given the low levels of asymptomatic parasitaemia one would expect in this context, it is unlikely that these infections are contributing much to ongoing infection and therefore affecting our estimates, especially as we do estimate unobserved sources of infection in our approach. This has been added to the discussion (lines 241-258).*

**• In trying to untangle how peaks in Rc correspond to peaks in cases, are there any season trends in Rc and cases in neighboring malaria endemic countries/areas that might help explain these season trends in Yunnan?**
 *This is an interesting point to consider. We do not have the individual level line lists required to estimate Rc in neighbouring malaria endemic countries (and in some of these contexts transmission may be too high for our methods to be appropriate), however monthly incidence in towns along the Myanmar-China border during 2012 were found to have a single peak which coincided or followed peaks in precipitation (1). This also reflects our findings for the peak in incidence. Both peaks in Rc are during the tail ends of environmental suitability for malaria transmission in Yunnan, which is from April- October.*

*We make the assumption that prior risk of unobserved case is constant throughout year (constant hazard function), however this may not be the case if there is more movement and importation at certain times of the year.*  *Therefore, this apparent bimodal seasonality in Rc estimates could be driven by the patterns of importation and changes in the risk of unobserved infections which we have not allowed for within our inference framework. An interesting direction for future work would be to interrogate this further with additional data about movement e.g. mobile phone data and/or antibody markers of exposure to malaria – however care must be taken not to introduce bias.*

**Reviewer #2**

**General

Line 59: provide more details about the malaria elimination plan. Did this include changes in prophylaxis or vector control? Has China done anything different compared to other countries to achieve success?**
*IRS was carried out and LLITNs were distributed but there were no major changes to interventions from this perspective. Stratification and the introduction of the 1-3-7 strategy has been a key part of the elimination plan and has begun to be adopted by other programmes due to the success the strategy achieved in China. These key aspects are summarised in lines* 59-63.

**Line 132: discuss probable sources of uncertainty.**
*The main sources of uncertainty were small data sizes (Bernoulli observations) and clustered spatial locations. Our Bayesian geostatistical model accounted for uncertainty when data were far from each other spatially, but also incorporated uncertainty from heterogenous observations.*

**Line 136: “almost certainly due to the smaller sample size” —> remove “almost certainly” or suggest other reasons.**

*done*

**Methods

Line 305: should j and i be reversed, e.g. case i infecting case j?**
*yes – now corrected*
**Line 321: briefly describe what a Rayleigh distribution is**

*done*

**Line 334: define SI on first mention**

*done*
 **Line 309: provide more details about ref 20.**
*done*

**Line 359: note different reference style.**
*done*
**Line 362: is the backslash symbol intended?**

*Yes, here this refers to a set difference. It is standard in set builder notation. In this equation (*$t_{1},\ldots,t_{n}\backslash t_{i})$ *means all the times of symptom onset which are not* $t_{i}.$

**Geostatistical model specifics

How was the geospatial model spatially and temporal structured? Include space-time indicators/explanations where relevant. What are the assumptions about chosen environmental covariates and R_c? explain. Show parameter estimates for covariates.**

*The model covariates were spatially structured at 2.5 arc minutes - roughly 5x5km grid squares, and the data were coordinate references. The Integrated nested laplace approximation (INLA) was used to estimate covariate coefficients and generate (approximate) posterior estimates of risk across a 5x5km raster. The spatial model itself was a gaussian markov random field mode (GMRF)l. We assumed a binomial likelihood for Rc>1 with functions modelled as a GMRF (with both mean and covariance function). Each year was analysed separately, and there was temporal structure in the temperature covariates, which were dynamical monthly variables. We have the full mathematical description of the model in the text.*

*We chose covariates which have previously been found to be strongly mechanistically linked to malaria transmission due to their impact on vector habitat: temperature and its variability (night, day, diurnal difference) , precipitation, elevation and accessibility/urbanicity. These covariates have also been found to predict malaria prevalence (2). We have added additional description outlining the information above and updated the covariate table as well as adding a table summarising the posterior parameter estimates for the covariates.*

**Figures and Table

Figure 2: change legend title from imported to ‘source of infection’ (or something along those lines), change blue label to ‘locally acquired’ and red label to ‘imported’.**
*done*

**Figure 5: use different line type for 2.5 and 97.5 percentile values.**

*done*

**Table 1: include spatial and temporal resolution of data. Provide more details for data source, including references. Capitalise first letter.**

*done*

**Non-exhaustive list of typos/suggested edits:**

*Thanks, all corrected*

**Line 80: it can be unclear whether —> it is unclear if**

*done* **Line 119:** **0.5 <= epsilon <= 0.8**

*done* **Line 129: missing fullstop after (Figure 2)**
*done*

**Line 151: and —> an**
 *done*

References

1. Hu Y, Zhou G, Ruan Y, Lee MC, Xu X, Deng S, Bai Y, Zhang J, Morris J, Liu H, Wang Y. Seasonal dynamics and microgeographical spatial heterogeneity of malaria along the China–Myanmar border. Acta tropica. 2016 May 1;157:12-9.
2. Bhatt S, Weiss DJ, Cameron E, Bisanzio D, Mappin B, Dalrymple U, Battle KE, Moyes CL, Henry A, Eckhoff PA, Wenger EA. The effect of malaria control on Plasmodium falciparum in Africa between 2000 and 2015. Nature. 2015 Oct;526(7572):207.

Best wishes,

Isobel Routledge
